# DNA Familial Binding Profiles Made Easy: Comparison of Various Motif Alignment and Clustering Strategies

**DOI:** 10.1371/journal.pcbi.0030061

**Published:** 2007-03-30

**Authors:** Shaun Mahony, Philip E Auron, Panayiotis V Benos

**Affiliations:** 1 Department of Computational Biology, School of Medicine, University of Pittsburgh, Pittsburgh, Pennsylvania, United States of America; 2 Department of Computer Science, Faculty of Arts and Sciences, University of Pittsburgh, Pittsburgh, Pennsylvania, United States of America; 3 Department of Biological Sciences, Duquesne University, Pittsburgh, Pennsylvania, United States of America; 4 Department of Molecular Genetics and Biochemistry, School of Medicine, University of Pittsburgh, Pittsburgh, Pennsylvania, United States of America; 5 Department of Human Genetics, Graduate School of Public Health, University of Pittsburgh, Pittsburgh, Pennsylvania, United States of America; 6 University of Pittsburgh Cancer Institute, School of Medicine, University of Pittsburgh, Pittsburgh, Pennsylvania, United States of America; Washington University, United States of America

## Abstract

Transcription factor (TF) proteins recognize a small number of DNA sequences with high specificity and control the expression of neighbouring genes. The evolution of TF binding preference has been the subject of a number of recent studies, in which generalized binding profiles have been introduced and used to improve the prediction of new target sites. Generalized profiles are generated by aligning and merging the individual profiles of related TFs. However, the distance metrics and alignment algorithms used to compare the binding profiles have not yet been fully explored or optimized. As a result, binding profiles depend on TF structural information and sometimes may ignore important distinctions between subfamilies. Prediction of the identity or the structural class of a protein that binds to a given DNA pattern will enhance the analysis of microarray and ChIP–chip data where frequently multiple putative targets of usually unknown TFs are predicted. Various comparison metrics and alignment algorithms are evaluated (a total of 105 combinations). We find that local alignments are generally better than global alignments at detecting eukaryotic DNA motif similarities, especially when combined with the *sum of squared distances* or *Pearson's correlation coefficient* comparison metrics. In addition, multiple-alignment strategies for binding profiles and tree-building methods are tested for their efficiency in constructing generalized binding models. A new method for automatic determination of the optimal number of clusters is developed and applied in the construction of a new set of familial binding profiles which improves upon TF classification accuracy. A software tool, STAMP, is developed to host all tested methods and make them publicly available. This work provides a high quality reference set of familial binding profiles and the first comprehensive platform for analysis of DNA profiles. Detecting similarities between DNA motifs is a key step in the comparative study of transcriptional regulation, and the work presented here will form the basis for tool and method development for future transcriptional modeling studies.

## Author Summary

Transcription factors are primary regulators of gene expression. They usually recognize short DNA sequences in gene promoters and subsequently alter their transcription rate. It is known that structurally related transcription factors often recognize similar DNA-binding patterns (or *motifs*). Comparison of these motifs not only provides insights into the evolutionary process they undergo, but it also has many important practical applications. For example, motifs that are found to be “similar” can be combined to form generalized profiles, which can be used to improve our ability to predict novel DNA signals in the promoters of co-expressed genes, and thus facilitate a more accurate mapping of gene-regulatory networks. However, to date there is no comprehensive platform that will allow for an efficient analysis of DNA motifs. Furthermore, the efficiency of the methods used to assign similarity between DNA motifs has not been thoroughly tested. This paper takes an important first step towards this goal by evaluating available comparison strategies as applied to DNA motifs and by generating an improved familial profile dataset.

## Introduction

Transcription factor (TF) proteins usually recognize a small number of DNA targets via the formation of sequence-specific and nonspecific molecular interactions. Understanding the evolution of TF DNA-binding preferences will not only provide useful insights on the mechanism of DNA recognition, it will also allow more accurate prediction of genomic regulatory elements, which still constitutes a major hurdle in understanding cellular gene regulatory networks. Furthermore, high-throughput studies, such as microarray and ChIP–chip, generate a number of DNA motifs that are putative targets of usually unknown TFs. In this study, we present an alignment and comparison platform that is optimized for DNA motifs, thereby allowing for their efficient analysis and enabling and formalizing their evolutionary study. This platform is called *STAMP*, for similarity, tree-building, and alignment of DNA motifs and profiles.

**Figure 1:**
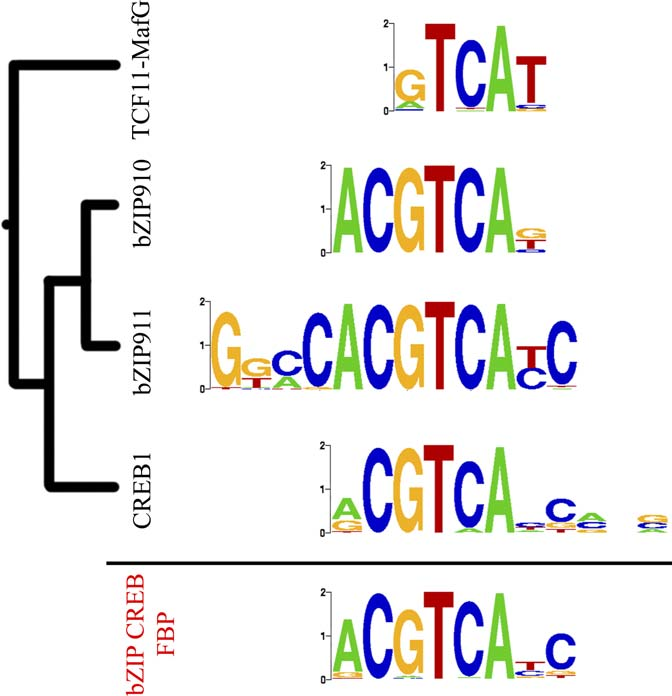
Illustration of Familial Binding Profile Construction In this example, the binding motifs for four bZIP–CREB transcription factors are aligned in a multiple-motif alignment. The generalized familial binding profiles correspond to the weighted average of the individual profiles.

TF DNA-binding preferences are usually modeled via frequency matrices, derived from alignments of known sites (see Methods). Typically, these *position-specific scoring matrices* (PSSMs) assume independency between the base positions [1]. It has been recognized that structurally related TFs often share similarities in their DNA-binding motifs, although the extent to which this happens depends on the TF family [2–5]. Generalized binding models or *familial binding profiles* (FBPs), a term coined by Sandelin and Wasserman [6], constitute an “average” binding specificity of a family of TFs (see Figure 1 for an illustration of the FBP concept). FBPs can be incorporated in pattern-finding algorithms as prior knowledge in order to bias them towards motifs from a particular TF family [6–8]. This is useful if the investigator expects to find motifs from a particular class of TFs. The use of FBPs as prior information focuses the motif search on biologically relevant patterns, offering a way to improve upon the currently limited performance of DNA motif-finders [9]. FBPs have been used to infer the identity of the TF family bound to predicted novel motifs [6,8,10], and to remove degeneracy between related motifs in the motif repositories [11–13]. More recently, FBPs have been used to help estimate the binding specificity of regulatory proteins from ChIP–chip data [14].

The early studies introducing FBPs demonstrated their potential in regulatory DNA analysis. However, the methods employed to compare and align DNA-binding motifs, a key aspect in constructing FBPs, have not been thoroughly studied. Currently, the construction of FBPs is based on (semi)-empirical clustering methods and ad hoc distance metrics. Ungapped local motif alignments [6,7] or enumeration of subsequence frequencies across related motifs' members [10,11] are typically used to compare PSSMs, although it is not yet known if these strategies are optimal. Even the definition of binding motif families and subgroups is currently problematic. Structural information and protein sequence comparisons have been previously used to guide manual clustering of TF binding profiles [6,8], although automatic methods have been recently introduced [7].

For more reliable FBP construction methods, and in order to expand the area of applications for generalized binding models, a detailed evaluation of a variety of motif alignment strategies is required. Motif evolution and the motif dependence on the proteins' structural properties need to be investigated. Sandelin and Wasserman [6] did an important first step when they created a set of 11 FBPs for the nonzinc finger families. During the FBP construction, they noticed that the bZIP family exhibited two different DNA-binding patterns, so they partitioned it into CCAAT/enhancer binding protein (C/EBP)- and cAMP response element-binding (CREB)-related proteins. However, their FBP clustering was done manually and other (sub)family characteristics may have been missed. On the other side of the spectrum, two families may have similar binding preferences, and although they may not belong to the same structural group, it may be reasonable to cluster their binding profiles together, so that the overall number of false positive predictions is reduced. Finally, a detailed analysis of the structural properties of the protein–DNA complexes, together with automatic clustering and classification results, is hoped to shed more light on the evolution of the DNA preferences and their utility in prediction and classification studies.

Schones et al. have compared the effectiveness of three profile distance metrics [13]. We expand their study by evaluating combinations of six distance metrics, three pairwise alignment methods, two multiple-alignment strategies, and two tree-building algorithms. In addition, we develop a new statistic for automatically deciding the optimal number of clusters in a given motif tree. We use this statistic on the tree obtained from the optimal distance metrics and alignment strategies combination to generate a new set of FBPs without prior knowledge of TF structural classification. The new collection of FBPs exhibits better TF classification accuracy than previous manually derived clusters [6] and identifies similarities and differences in the binding preferences of TF (sub)families.

Although species-specific binding preference may emerge for some TFs [15], in general structurally related TFs often share similarities in their DNA-binding preferences. Exploring this trend, Narlikar and Hartemink built a Bayesian TF structural family classifier based on the DNA motifs [10]. We found that the same accuracy of this sophisticated method can be achieved with simple motif similarity searches when the appropriate alignment algorithms are used. Correctly predicting the TF structural class for novel motifs will be a crucial step in the interpretation of experiments that aim to systematically estimate regulatory motifs in entire mammalian genomes (e.g., [16]).

## Results

### Distributions of Similarity Scores in PSSM Columns from Known TFs

**Figure 2:**
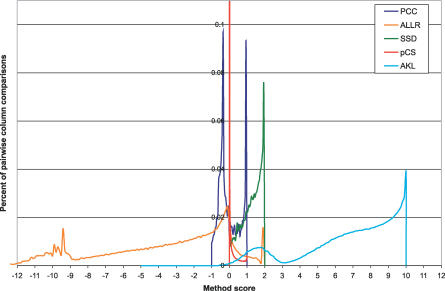
Distribution of the Observed Scores of Column-to-Column Comparisons for the Five Main Similarity Metrics Columns are obtained from the TRANSFAC database [17]. The ALLR_LL distribution is identical to ALLR for every point ≥2 (unpublished data). Comparison of the JASPAR motif columns yielded similar results.

All columns from the known PSSM models in the TRANSFAC database [17] were compared with each other using the six metrics presented in Table 1 (see Methods). Figure 2 shows the great variability in the range and distribution of the scores. Both *Pearson's correlation coefficient* (PCC) and *average log likelihood ratio* (ALLR) have negative expected values, which makes them especially suitable for use in alignment algorithms (although, negative mean values can be obtained from any metric by subtracting an appropriate number). Three of the methods, namely PCC, *sum of squared distances* (SSD), and *p-value of chi-square* (pCS), have peaks of very small variance, with PCC having two distinct peaks. Comparison of JASPAR [18] columns gave similar results (unpublished data).

**Table 1:**
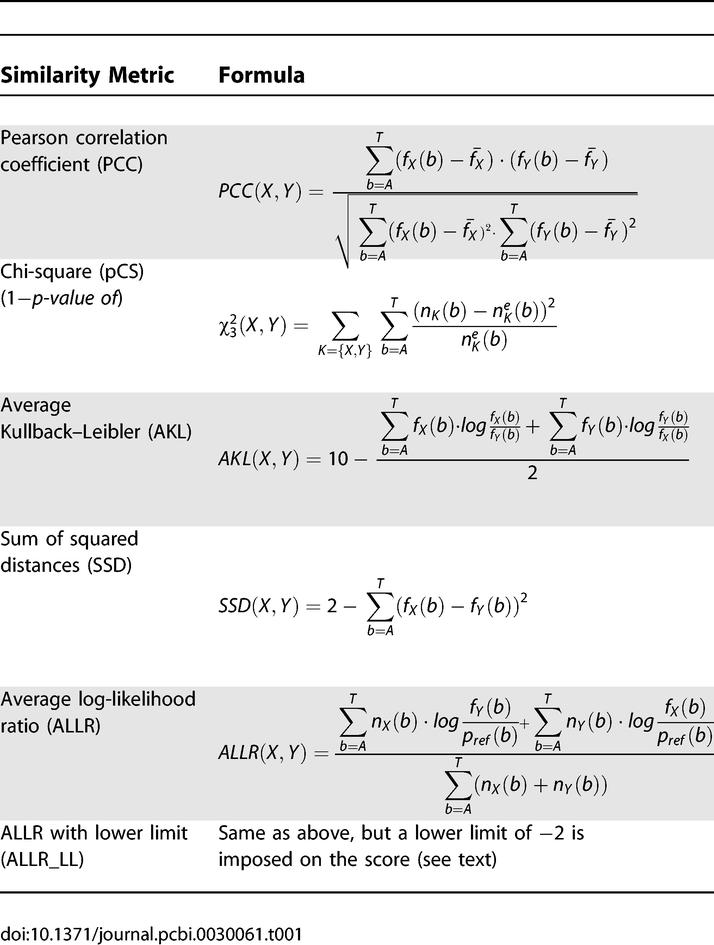
The Six Similarity Metrics Used in This Study for PSSM Column Similarity and Motif Alignments

### Evaluation of the Similarity Metrics in PSSM Column Comparisons

**Figure 3:**
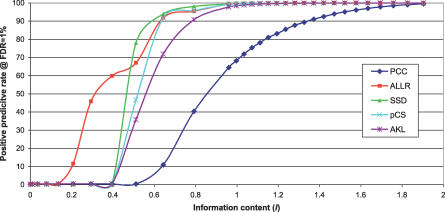
Performance of the Five Main Similarity Metrics in Discriminating between Columns Sampled from Dirichlet Distributions around Information Content *I* and a Background Distribution The plot shows the positive predictive rate for an FDR of 1% as a function of the information content.

Each of the six metrics was tested for its ability to discriminate between columns randomly sampled from two distinct distributions: an information content–specific distribution and a background (reference) distribution. For information content, *I,* Figure 3 plots the percent of the 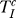 -sampled columns that were included in the area around 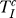 when a *false discovery rate* (FDR) of 1% was reached. For lower information content, ALLR and SSD perform best at discriminating columns sampled around 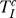 and 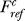 . The finding that ALLR is a better discriminator than pCS metric may seem to contradict the findings of Schones et al. [13]. However, apart from our sampling size being larger, their evaluation focused on the whole motif level. As we will see later, the advantageous performance of ALLR in column-to-column comparisons does not seem to extend to motif-to-motif comparisons.

### Comparing Motif Alignment Strategies: The “Best-Hit” Evaluation

It is difficult to construct an unbiased artificial dataset for the evaluation of motif alignment strategies. However, an indication of performance may be gained from similarity searches of a motif against all motifs in a database. Generally, in databases with good representation, the best match to a given motif is expected to be a motif associated with a member of the same structural class [6]. The “best-hit” approach can be used to assess the relative effectiveness of column-scoring metrics and alignment-method combinations by finding the proportion of motifs that match another member of the same structural class using each strategy.

One hundred and five combinations of similarity metrics, alignment methods, and gap penalty values were tested over two datasets: the JASPAR- and TRANSFAC-derived models (see Methods). The top 15 and bottom 15 performing strategies/combinations are presented in Table 2, and a full table of results is available in Table S1. Smith–Waterman local alignments populate the list of best performing results, indicating that they are generally better than Needleman–Wunsch global alignments in motif alignment applications. The results also suggest that the PCC and SSD metrics are on average more effective than the AKL, pCS, and ALLR metrics (including ALLR_LL) for whole-motif comparison. The best-performing combination is Smith–Waterman local alignment using the SSD metric and gap open = 1 (average accuracy 0.811), whereas Smith–Waterman local alignment using the PCC metric also scores highly (seventh-best score; average accuracy 0.805). The first strategy that uses the AKL metric appears at position 28 of the list, and the first strategy using ALLR_LL appears at position 37. Strategies using the standard ALLR metric first appear at position 48, and strategies using the pCS metric first appear at position 60.

**Table 2:**
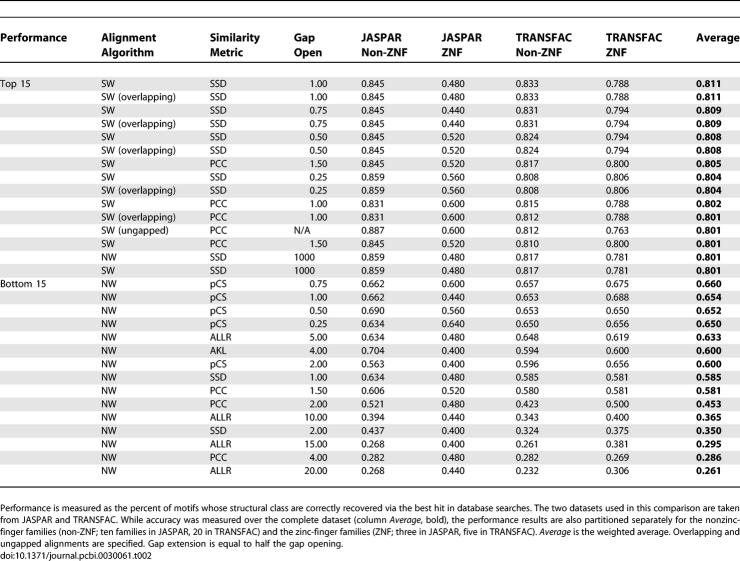
The Top 15 and Bottom 15 Performing Alignment Strategies

### Predicting the TF Structural Class from its Binding Preferences

In the study of Narlikar and Hartemink [10], a sparse Bayesian learning algorithm was used to predict the structural class of the TF that binds to a given DNA motif. The test dataset in that study consisted of the six largest motif families in TRANSFAC, and Narlikar and Hartemink's algorithm was able to correctly predict the TF structural family for 86% of these DNA-binding motifs. By analyzing the same dataset, we found that the best-hit approach (with ungapped Smith–Waterman alignment and the PCC metric) yields practically the same performance (87%). The best-hit searches perform better in predicting the bZIP, C4 zinc finger, and Forkhead families, whereas the Bayesian learning is better in the more diverse (in terms of DNA binding motifs) C2H2 zinc finger, homeodomain, and bHLH families (Table 3). We will later show that the appropriate clustering of DNA motifs can help improve the classification accuracy.

**Table 3:**
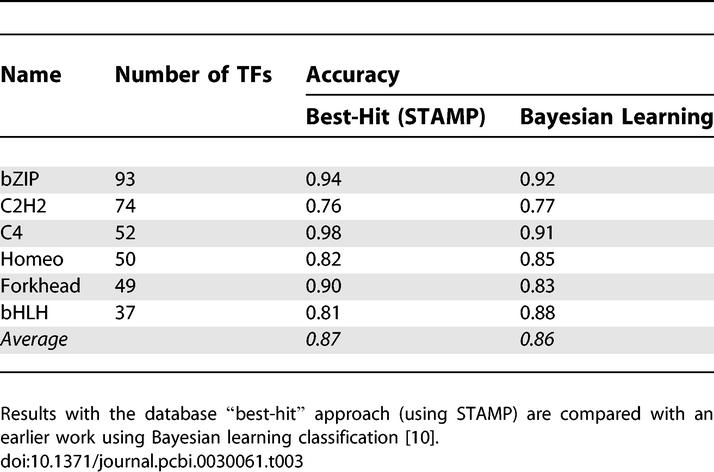
Performance of TF Structural Family Classification Based on DNA-Binding Preferences in the Six Largest Motif Families in TRANSFAC

### Performance of Motif Tree-Building Methods

**Figure 4:**
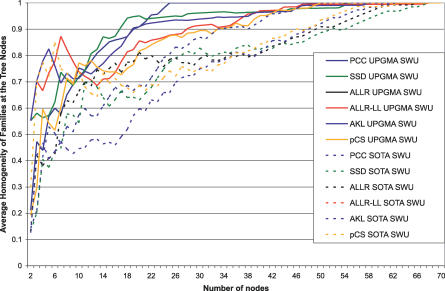
Average Homogeneity of Families Represented at Each Tree Node as a Factor of the Growth of the Tree Six scoring metrics and two different tree-building methods are tested with ungapped Smith–Waterman alignments.

**Figure 5:**
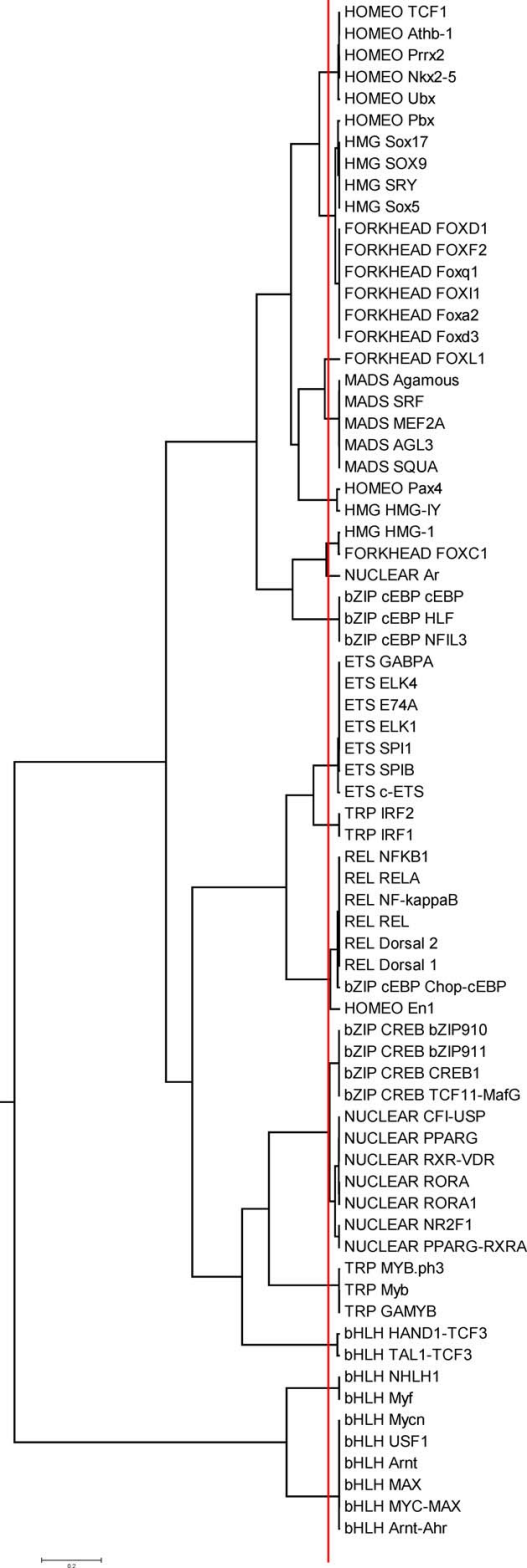
The Tree Resulting from a UPGMA Tree Construction of Ten JASPAR Families (71 Motifs Total) Using the PCC Scoring Metric and Smith–Waterman (Ungapped) Alignment Method The red line represents the level at which the *CH*_log_ metric estimates the optimal number of data clusters on the tree.

Sandelin and Wasserman manually constructed FBPs for those ten nonzinc-finger structural families for which four or more motifs exist in the JASPAR database (71 motifs) [6]. One of the ten families, bZIP, produced two distinct FBPs: one related to C/EBP and one related to the CREB proteins. The set of 71 profiles provides an appropriate dataset for testing tree-building strategies and automatic clustering methods. In this study, an agglomerative (UPGMA) and a divisive (SOTA) strategy were compared (see Methods). The most effective alignment strategy for the nonzinc-finger JASPAR dataset, the ungapped Smith–Waterman alignment, was used with each of the six similarity metrics in conjunction with UPGMA or SOTA. Performance was measured as the *average homogeneity* of the families at each leaf node on the tree and with respect to the tree growth. For a given node, a performance average homogeneity score of 1 denotes that only one family is represented in the motifs clustered at that node (perfect homogeneity); a score of 0.5 denotes that two equally represented families are clustered in that node; etc. The point at which the average homogeneity of the leaf nodes reaches 1 is the point where motifs have been successfully separated on the basis of structural class (although a class might be split into multiple nodes). As can be seen in Figure 4, UPGMA generally performs better than the neural tree method (SOTA), regardless of the similarity metric. Ungapped Smith–Waterman alignment using the PCC metric is by far the most successful metric on this dataset, managing to separate all ten structural families (100% average homogeneity) with only 25 leaf nodes. In addition, ungapped Smith–Waterman alignment using the SSD metric achieves 95% average homogeneity with 26 leaf nodes. The actual tree resulting from the combination of ungapped Smith–Waterman alignment, the PCC metric, and the UPGMA tree construction method displays a high degree of separation of the TF structural classes (Figure 5).

### Automatic Construction of Familial Binding Profiles

Estimating the optimal number of data clusters on a tree of binding motifs is of significant interest in classification and familial binding property analysis applications. It is well-known, however, that this is an inherently arbitrary procedure; different criteria on where to “split” the tree will give different estimates of cluster number. A number of statistics have been described that aim to estimate the optimal number of clusters (e.g., [19–21]), usually by seeking an optimal balance between intercluster and intracluster variability.

**Figure 6:**
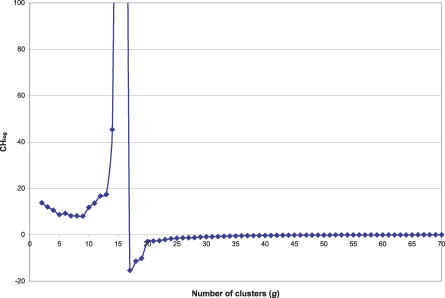
The Behaviour of the Calinski and Harabasz–Based Log-Metric (*CH*_log_) for the Tree in Figure 5 as the Number of Clusters (*g*) Is Varied The value of *g* = 17 produces a global minimum in the value of *CH*_log_.

We used a subset of the JASPAR motifs to understand how different metrics perform in determining the optimal number of clusters in a tree. For this purpose, only closely related members of the ETS, REL, Forkhead, high mobility group (HMG), and MADS families were used. The statistics we compared were: the Gap statistic of Hastie, Tibshirani, and Walter (*HTW*) [22], the standard Calinski and Harabasz (*CH*) [19], and a derivative (*CH* **_log_**) we developed (Table 4). When tested on the tree of the five well-defined families, the standard *CH* statistic didn't yield any local maximum number of clusters. We believe this is because this statistic performs well when the number of clusters is small compared with the number of points. Otherwise, the within-cluster difference goes quickly to zero, driving the *CH* to infinity. The *CH*_log_, by design, avoids this problem. *HTW* and *CH*_log_ both gave the correct optimal number of clusters in the five-family dataset. When tested on the full nonzinc-finger JASPAR dataset (71 motifs) (Figure 5), however, *HTW* and *CH* yielded no optimum number of clusters, whereas *CH*_log_ gave 17 (Figure 6), which is a reasonable number (compared with 11 of Sandelin and Wasserman). For the most part, the identified clusters of motifs seemed reasonable both in terms of binding motif conservation (manual examination) and in terms of TF subfamily classification. More information is provided in the Discussion section. The suitability of *CH*_log_ was also tested in trees with two, three, four, and six well-defined clusters (from the above set of JASPAR PSSMs) and always yielded the correct answer.

**Table 4:**
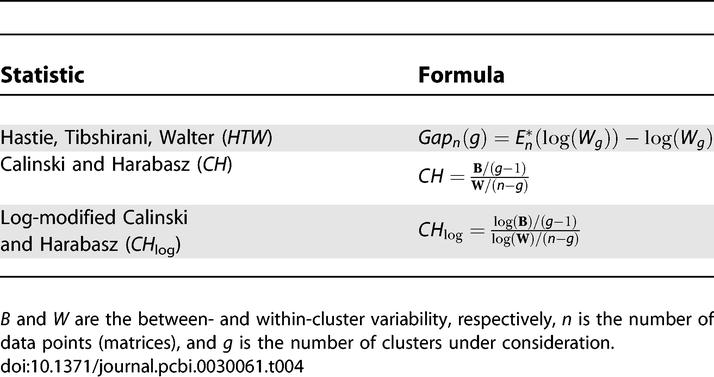
The Four Statistics Tested on Automatic Clustering of the 71 JASPAR Motifs

We test the hypothesis that the 17 automatically generated FBPs are a more accurate representation of motif diversity in the JASPAR set than the 11 manually constructed motifs using *leave-one-out cross-validation* (LOOCV). In this test, we treat the set of FBP clusters as static multiple alignments and remove the contribution of each motif from its appropriate FBP in turn. The removed motif is then compared against all regenerated FBPs, and treated as correctly classified if it most closely matches the FBP from which it was withdrawn. LOOCV using Sandelin and Wasserman's 11 manually defined FBPs results in nine misclassifications, or a classification performance of 62/71 = 87% (this performance rate was also reported in [6]). By comparison, in our dataset of 17 automatically defined FBPs, LOOCV resulted in two misclassifications, which, combined with the two unclassifiable singleton clusters, suggests a classification performance of 94% (67/71).

**Figure 7:**
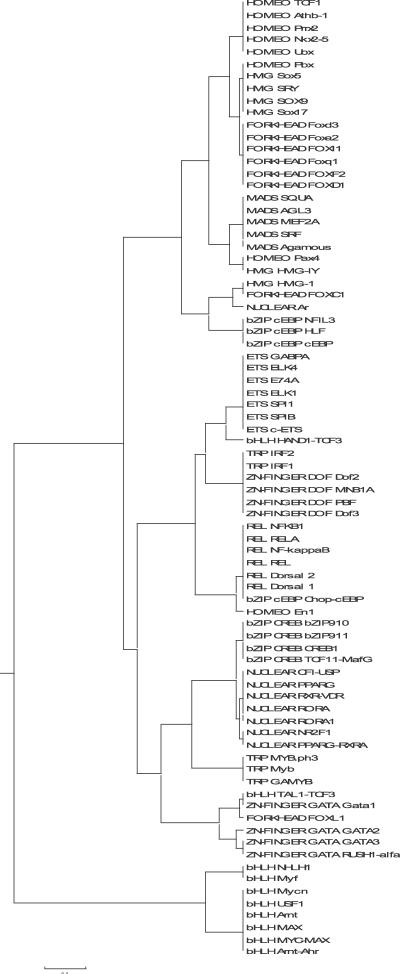
The Tree Resulting from a UPGMA Tree Construction of 12 JASPAR Families (79 Motifs Total) Using the PCC Scoring Metric and Smith–Waterman (Ungapped) Alignment Method This tree includes the two zinc-finger families (GATA and DOF).

JASPAR also contains three zinc-finger motifs that were not used in the Sandelin and Wasserman FBP construction. One of them, C2H2, includes TF proteins with highly divergent patterns of contacts (see Discussion). The other two, DOF (a C4 zinc-finger family) and GATA, have quite conserved DNA-binding patterns. We repeated the above analysis by including the DOF and GATA motifs in JASPAR (four motifs in each family). Our method determined 18 clusters (including two singletons), and a LOOCV test resulted in 72/79 correct classifications (91%). Sandelin's and Wasserman's method on the 13 clusters (the previous 11 and the two zinc fingers) resulted in 60/79 correct classifications in the LOOCV test (76%). Compared with the 71-motif tree (Figure 5), the new tree is identical in 15 of the clusters (Figure 7). The main difference is that the two-member cluster of the heterodimeric bHLH proteins, TAL1-TCF3 and HAND1-TCF3, is now split. The TAL1-TCF3 motif is part of a new cluster with the (previously singleton) FOXL1 and the GATA-1 zinc-finger protein motif. The remaining three GATA proteins formed a new cluster. All four DOF proteins are co-clustered with the IRF proteins.

### The STAMP Platform

All described methods have been compiled in a software platform (STAMP) (Mahony S, Benos PV, STAMP: A web tool for exploring DNA-binding motif similarities, unpublished). STAMP is modularly designed to allow any combination of column–column scoring metric, alignment method, tree-building algorithm, and multiple-alignment strategy to be used. Its potential uses range from simple motif database searches to identify the TF that may bind to a particular motif to a full-scale analysis of multiple-aligned genomic regions. In the section below, examples of both these uses are provided. STAMP is publicly accessible from http://www.benoslab.pitt.edu/stamp/.

## Discussion

### Comparison of PSSM Column Distance Metrics and Alignment Strategies

Six PSSM column similarity metrics were evaluated together with three pairwise alignment methods (two gapped and one ungapped), two multiple-alignment strategies, and two tree-building strategies on motif datasets. The results showed that the Smith–Waterman local alignment algorithm used with the PCC or SSD metrics generally performs better in aligning the currently available PSSM models with models for which the associated TF belongs to the same structural family. We also discovered that the high efficiency of some metrics in column-to-column comparison does not extend to the alignment of whole motifs, which is a surprising and previously overlooked outcome. In the case of the ALLR metric, we believe that this inconsistency is due to the metric's very negatively skewed scoring distribution (Figure 2). Although such a distribution might be advantageous in distinguishing between PSSM columns with low information content, it also makes motif alignment more difficult, especially when the motifs contain low-scoring regions that can rigorously negate the overall score. The difficulties in using ALLR became more apparent with the Needleman–Wunsch global alignment, as low scores that are frequently observed in the beginning and/or at the end of the alignment could not be adequately subsidized by the positive scores in the alignable areas. The use of the alternative ALLR_LL metric (i.e., ALLR with a lower limit of −2) improved the results slightly. However, ALLR is the only metric that takes into consideration the background distribution when it compares two columns, which, in combination with the fact that it distinguishes better between columns of low information content (Figure 3), can be advantageous in identifying the correct PSSM model among closely related models (e.g., those belonging to the same family).

Smith–Waterman local alignments were found to be more effective than Needleman–Wunsch global alignments for DNA motifs. This is expected given the current status of the motif databases. Motifs in existing databases usually result from some automated method that runs on a set of unaligned sequences recorded in these databases. These motifs frequently consist of a “core” area of columns with high information content, surrounded by columns of low(er) information content. On such motifs, local motif alignment methods will tend to perform better than global alignment methods. Structurally, this also makes sense, since binding sites are often recognized by a single structural sequence recognition element (e.g., α-helix) either surrounded by or adjacent to a less-specific element that provides additional binding energy. Interestingly, ungapped algorithms or gapped algorithms with high gap-opening penalties generally performed better with the same metric (Table 2). This is probably due to the fact that the motifs for TF families in JASPAR and TRANSFAC share ungapped regions of similarity. However, the difference between ungapped/high penalty and gapped with lower-gap penalty algorithms is marginal in the JASPAR and TRANSFAC databases. Gapped alignment methods are expected to be more effective when aligning families of motifs that share common half-sites with variable length spacer regions, like many prokaryotic sites.

### Differences in Classification Efficiency between TF Structural Families

An unexpected finding was that the current multiclass motif classifiers perform no better than simple best-hit similarity queries against a motif database *when appropriate motif alignment methods are used*. Interestingly, the two zinc-finger families (C2H2 and C4) are predicted with the worst and the best efficiency, respectively, which reflects their binding geometries. The C4 factors form extensive networks of contacts along the length of an α-helix embedded in a B-DNA major groove. As a result, their target sequences are very conserved and thus their predictions easier. The C2H2 zinc fingers on the other hand contact the DNA helix at an angle using only few amino acid side-chains extending from the end of a less intimately associated helix. This results in less binding dependence upon individual amino acid sequences. Changes in certain “key” amino acid positions can drastically alter the DNA-binding specificity, thus yielding highly variable targets.

bZIP factors bind to DNA as dimers in palindromic targets and they select individual half-sites in the process. The monomers readily dissociate from the dimeric form, binding DNA initially as half-site monomers [23]. This kinetic selection process, along with the intimate association of the recognition helix with the major groove (similar to C4 Zn fingers) likely provides exquisite selectivity. The same selectivity may not be realized for the bHLH proteins. Although these factors also select out half-sites, their monomers, in contrast to bZIPs, have very low dissociation rates and act more like covalently linked DNA-binding domains (similar to C2H2 factors). Also, the angle of interaction between the recognition helix and the major groove is more obtuse for bHLH than for bZIP (23 versus 20 degrees), resulting in less interaction with half-sites (3 bp instead of 4 bp). These differences may explain why a bHLH behaves more like a C2H2 in target heterogeneity.

The relatively poor prediction specificity exhibited for the Homeo HTH domain proteins stems from the binding of these factors to highly divergent targets (usually recognizing mostly an “AT” motif), probably due to their dependence upon partner domains that are either dissociable (like Hox-Pbx, Ubx-Exd, and Mat a1-alpha2) or covalently linked (like Oct, Pax, and Pit). Member families of the monomeric wHTH subtype have strong consensus correlations (like the ETS and Forkhead families with consensus GGAA and TAAACA, respectively). This results in higher prediction efficiency for Forkhead than for the more variable Homeo class. It is this idea of variability, perhaps dependent upon multimerization-related constraints, that may be a useful basis for the distinctions we observe.

Another problem that may impose a limit in the classification efficiency of any method (regardless of the TF family) is the quality of the TFBS alignment and the resulting PSSM models. It is hoped that with the accumulation of new data, this will become less of a problem in the future.

### Clustering of DNA Profiles: Automatic FBP Construction

Sandelin and Wasserman [6] had previously built 11 FBPs from 71 nonzinc-finger PSSM models (ten TF families) available in the JASPAR database. Their manual clustering of the PSSM models was based on prior knowledge of the structural class of the corresponding TF. An exception to this general rule was the bZIP family, for which they constructed two FBPs (CREB- and C/EBP-related) after observing very different DNA patterns. The FBP corresponding to each structural class was calculated from a multiple-motif alignment where the contributions from outlying motifs were negatively weighted. The 11 familial binding profiles and the 71 motifs in the training set are available to view from the JASPAR database website (http://mordor.cgb.ki.se/cgi-bin/jaspar2005/jaspar_db.pl). Sandelin and Wasserman's manual approach is suitable for relatively small sets of binding motifs where the structural class corresponding to each motif is known, and where the representatives from each component structural class bind a set of closely related target motifs. However, in the more general case, where families that bind diverse target motifs are included or where the structural class of certain motifs is unknown, it may be useful to attempt automatic generation of the appropriate familial binding motifs.

We developed a fully automated method for PSSM clustering, based on the combinations of metric, alignment strategies, and tree building examined in this study. The advantages of the automatic clustering are obvious. By remaining ignorant to the prior knowledge of the structural class of each motif, we can find cases where motifs from diverse structural classes are more suitably grouped together, if they have similar binding preferences. Similarly, the automatic approach avoids the temptation of forcing together subfamilies of the same structural class with different binding preferences. Also, “outlier” PSSMs can be easily detected through an automatic clustering and subsequently be excluded from the FBP.

**Figure 8:**
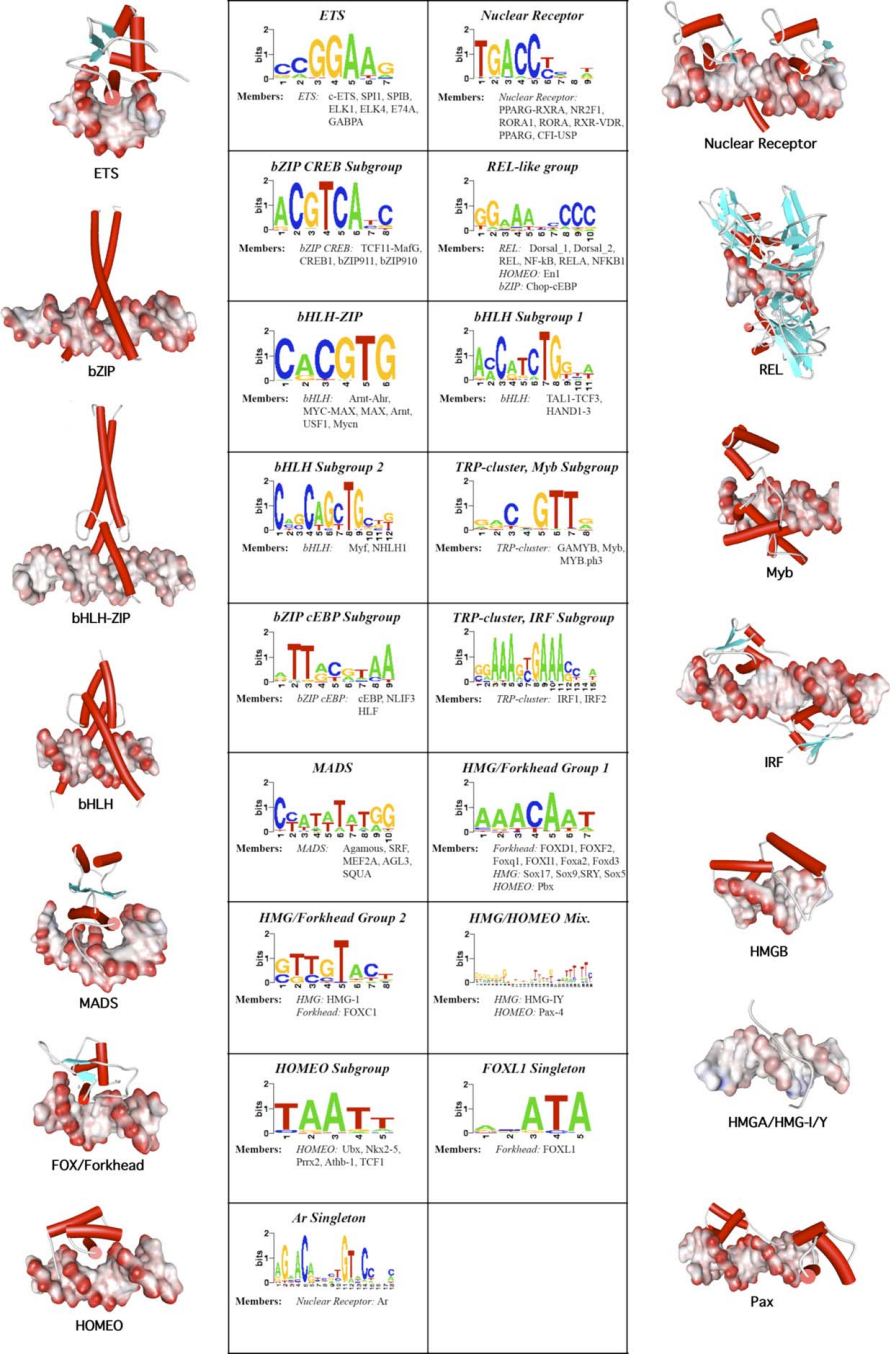
Optimal Number of Clusters of the 71 JASPAR Motifs, According to Our Method PCC with Smith–Waterman ungapped alignment was used as a scoring function. Examples of protein–DNA complexes are provided for comparison.

The method we used to determine the optimal number of clusters is similar to the Calinski and Harabasz statistic [19], but the intercluster and intracluster variability is calculated on the log-scale. We found this method to compare favourably with other methods on datasets with a known, well-defined small number of clusters and in the whole dataset. When applied on the 71 JASPAR PSSM models of our dataset, this method yielded 17 clusters, two of which are singletons and another two of which contain a pair of heterologous TFs each (Figure 8). Overall, the automatic clustering method divides the dataset into homogeneous clusters with respect to the structural group of the corresponding TF (note that the clusters are based solely on the *binding preferences* of the TFs). This agrees with the general notion that structurally similar TFs tend to have similar binding specificities. The MADS domain proteins and the wHTH ETS proteins are two examples of TF families with very conserved DNA-binding preferences. Also, *homeobox* and *nuclear receptor* family clusters are homogeneous, although some members of these families can be found in other clusters. Interestingly, our algorithm split the patterns of the members of the (so-called) TRP family into Myb-related proteins and IRF proteins. This may not be surprising, since these two HTH-like proteins exhibit distinct DNA-binding geometries. Myb proteins contain three HTH domains, only one of which is involved in the target recognition (with reported consensus YAAC[G/T]G). The IRF family consists of wHTH proteins that bind as homodimers via nonpalindromic direct repeats [24] or as monomers cooperatively with other proteins, like ETS [25]. The IRF motif (Figure 8) contains a repeat of the commonly reported [A/G]NGAAA consensus, which we attribute to the homodimerization binding of these proteins.

Our algorithm also correctly recognized three subfamilies in the major bHLH family. The six members of the bHLH-zip subclass (e.g., USF1, MAX, etc.) are clustered together, whereas the remaining four “standard” bHLH proteins (Myf, NHLH1) and bHLH complexes (HAND1-TCF3, TAL1-TCF3) form two separate clusters. Examination of the FBPs of these clusters (Figure 8) shows clearly that binding preferences are substantially different, reflecting their corresponding mode of DNA recognition. The bZIP binding motifs were also automatically split into two clusters, identical to the (manually) classified JASPAR FBPs: one with the CREB-like and one with the C/EBP-like proteins. We note the striking similarity between the bZIP/CREB and the nuclear receptor binding patterns. Still, since the only base position they differ in is one of high information content, our clustering method was able to distinguish between the two patterns.

**Figure 9:**
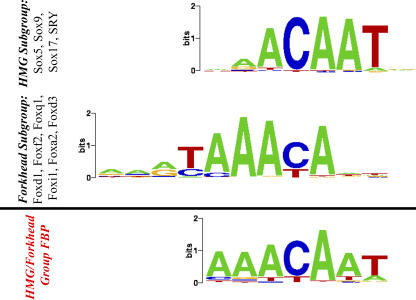
Similarity between the HMG and Forkhead Motifs These families are grouped together on the HMG/Forkhead Group I cluster (Figure 8).

The HMG proteins are represented by three protein families that bind chromosomal DNA. The two families represented in the JASPAR database are HMGA/HMGI/Y and HMGB/SOX/SRY, whereas the HMGN family is not represented. The HMGA proteins are members of the *AT-hook* family of TFs [26]. The HMGB proteins are structurally distinct *HMG-box* proteins [27]. Both families prefer to bind to AT-rich sequences in the minor groove with low selectivity. This is probably the reason that our algorithm clustered them together with the Forkhead family, which also binds to AT-rich sequences, but in the major groove. There is no structural similarity among these classes of proteins, and the mode of their interaction with the DNA suggests that the target similarity is coincidental. In fact, the two motifs are *not* identical, but they show significant overlap in four highly informative nucleotide positions (consensus: AACA) (Figure 9). Nevertheless, for those that use the FBPs for predicting the TF that binds to a given DNA motif, this provides an example where generating individual FBPs might lead to misclassification due to coincidental target similarity. Thus, for prediction purposes, we propose to keep these two families in the same cluster (FBP), since distinguishing between the two may be difficult (Figure 9). Notably, our algorithm identified another cluster composed of members of both families, suggesting there is a relationship between their motifs. Both these clusters contain members of the HMGB subgroup. The only member of the distinct HMGA subgroup, HMG-I/Y, clusters together with a homeodomain protein, Pax4. Pax proteins have two covalently linked HTH domains separated by a long linker and they also bind AT-rich sequences. The HTHs bind to 5-bp and 6-bp recognition sequences with an interpositioned 6-bp spacer that interacts with the linker [28]. This explains the long recognition sequence revealed in the FBP in this cluster.

When the two zinc-finger families were included in the analysis, the overall structure of the tree and the clusters remained the same, pointing to the stability of our multiple alignment and clustering algorithm. Most of the GATA proteins formed a new cluster, whereas all DOF proteins joined the cluster of the two IRF proteins. This is because the DOF consensus target sequence (AAAG) is part of the IRF motif (Figure 8). The cross-validation results in the extended family tree/clustering remained very high (91% compared with 94% in the smaller tree).

### The STAMP Platform

The STAMP platform, introduced in this study, contains all tested algorithms and can be efficiently used in BLAST-like searches against a database of PSSM models. Various datasets (including TRANSFAC and JASPAR motifs) are currently supported. In the future, it would be useful to incorporate other similarity metrics, alignment methods, and tree-building algorithms into the platform in order to allow for further exploration of optimal methods. Note, however, that it may not be possible to implement all of the known tree-building algorithms for motif alignment. Other distance-based methods (such as neighbour-joining [29]) rely heavily on additivity of the distance metric, which was not possible to define using our comparison metrics. Parsimony-based methods [30] rely on the estimation of substitution rates between sites, which is also not easily definable for frequency matrices. However, a substitution matrix has recently been defined for DNA-binding consensus sequences [31], so application of alternative tree-building methods may yet be possible in the DNA-binding motif domain (albeit not for PSSMs per se).

In summary, we expect that the methods and the results described in this study will facilitate the exploration of DNA-binding preference evolution amongst related transcription factors and will have a significant impact in many areas of gene research.

## Materials and Methods

### 

#### PSSM column-scoring metrics.

A PSSM model of length *L* is composed of a set of 4 × *L* weights (columns). Each column, *X,* follows a probability distribution, {*p_x_*(*b*)}*_b_*_∈{*A,C,G,T*}_, with the base-probability values reflecting the preference of the TF for the corresponding base in this position. The probability values can be estimated from the observed base counts {*n_x_*(*b*)}*_b_*_∈{*A,C,G,T*}_. We denote the estimated values *f*(X) = {*f_x_*(*b*)}*_b_*_∈{*A,C,G,T*}_. In practice, *p_X_* are estimated from *n_X_* plus some pseudocounts to reduce small-sample biases and to avoid zero probabilities. The assumption of independence between positions is not entirely accurate, but acts as a useful approximation [32–34]. In this study, only position-independent PSSM models are considered.

Six metrics (Table 1) are compared with respect to their efficiency in capturing similarities between PSSM columns and in aligning PSSM motifs. Let us suppose one wants to compare two columns ***X*** and ***Y*** *,* with total number of aligned sequences *N_X_* and *N_Y_* and (estimated) frequency distributions of *f̂*(*X*) = (*X_A_*, *X_C_*, *X_G_*, *X_T_*) and *f̂* (*Y*) = (*Y_A_*, *Y_C_*, *Y_G_*, *Y_T_*), respectively. We denote *X̄* and *Ȳ* the average frequencies in the two columns.

*Pearson Correlation Coefficient (PCC).* PCC is a popular similarity metric that has been used by us and others in comparing DNA models [7,32,35]. PCC gives a measure of agreement between two (unweighted) sets of observations by means of their covariance. Pietrokovski [36] found PCC to be the most effective among four metrics for protein profiles.

*p-Value of chi-square test (pCS).* Schones et al. [13] used the chi-square statistic as an approximation of the Fisher–Irwin exact test to investigate whether two columns are samples from the same multinomial distribution. In Table 1, *n_K_*(*b*) in the pCS formula represents the observed count of base *b* in column *K* (*K* ∈ {***X***, ***Y***}) plus one pseudocount. The expected occurrence of base *b* in column *K* is given by 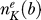 = (*N_K_*·*N_b_*)/*N*, where *N_K_* is the total number of counts in column *K, N_b_* is the total number of counts for base *b* in the two columns, and *N* is the sum of counts in both columns. The *p*-value is calculated from the 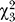 statistic and subtracted from 1 to yield a similarity score. We note that the pCS metric does not hold as an approximation of the Fisher–Irwin exact test when one or more bases has fewer than five observed counts in a column [13]. This condition occurs often in PSSM models.

*Average Kullback–Leibler (AKL).* The Kullback–Leibler distance (or *relative entropy*) is the weighted log-likelihood ratio distance between two distributions. The standard Kullback–Leibler is noncommutative and hence is not a true metric. However, it is frequently used in comparing TF–DNA preference probability distributions since it has an important quality: more weight is placed in the high-probability bases, where TF–DNA modeling algorithms need to be more accurate. Kullback–Leibler distance has been employed in the T-Reg Comparator motif similarity software tool and elsewhere [32,37,38]. In our study, we subtract the *average* Kullback–Leibler score from an arbitrarily defined maximum (10.0) to convert it to a similarity score. A theoretical maximum score cannot be defined for the AKL column distances, but more than 99% of the column-to-column scores in the TRANSFAC database [17] were found to have an AKL of less than 10.0.

*Sum of squared distances (SSD).* SSD is a simple scoring metric that was employed by Sandelin and Wasserman in their construction of FBPs [6]. Note that in this metric, the basic distance measure is subtracted from the maximum possible score (2.0) to convert it to similarity measure.

*Average Log Likelihood Ratio (ALLR and ALLR_LL).* The ALLR statistic was introduced by Wang and Stormo [31,39] as a way to measure similarity between two *informative* (i.e., different from the background) column distributions. Table 1 presents the formula for ALLR, where *n_K_*(*b*) is the observed count of base *b* in column *K* (*K* ∈ {***X***, ***Y***}) and *p_ref_*(*b*) is the background probability of base *b*. In this study, *p_ref_*(*b*) is assumed to be 0.25 for all bases. The ALLR metric has a skewed scoring distribution; the minimum score (≤−10) is much smaller than the maximum score (2). Therefore, we also tested a modified ALLR measure, termed *ALLR_LL,* in which the lower score was bounded by −2.

#### Comparing columns.

Information content–specific PSSM columns were constructed with *f_I_(C) = f_I_(G) = f_I_*(T) and *f_I_*(A) set such that the column information content had a specified value *I*. This column is denoted the “true” column centre, or 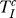 . One million columns were sampled independently from a Dirichlet distribution centered on 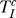 (Dirichlet *α* parameter = 20). The sum of nucleotide counts in each of the random columns was set to 30 to reflect the mean number of binding site instances used to construct TRANSFAC PSSMs. Another one million columns were sampled from a Dirichlet distribution centered on the zero information content column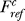 , where *f_ref_(A)* = *f_ref_(C)* = *f_ref_(G)* = *f_ref_(T)* = 0.25.

#### Comparing motifs of different lengths: *p*-Values.

To avoid length biases when comparing motifs of different lengths, we used the method of Sandelin and Wasserman for the calculation of empirical *p*-values based on simulated PSSMs [6]. Construction of a dataset of 10,000 simulated PSSMs was performed according to the instructions of Sandelin and Wasserman (http://forkhead2.cgb.ki.se/jaspar/additional) that reflected the properties of PSSMs in the JASPAR database.

#### Pairwise and multiple-motif alignment and tree-building methods.

*Pairwise motif alignment.* Needleman–Wunsch global alignment [40,41] and Smith–Waterman local alignment [42] were tested. Both methods allow for affine gap penalties. For this study, the gap-extension penalty is set to be half the value of the gap-opening penalty. Smith–Waterman alignments also allow the user to specify a minimum overlap length for the local alignment. In cases where we tested the performance of Smith–Waterman alignments with required overlap, the minimum overlap length was set to three columns. We also implemented an ungapped, extended Smith–Waterman alignment method, in which the “motif cores” of the PSSM models under comparison are aligned before extending the local alignment. A “core” is defined as the longest of (a) the four most informative adjacent columns; and (b) the “trimmed” motif (starting and ending at a position with information content of at least 0.3). The ungapped extended Smith–Waterman alignment was found to have advantages in aligning groups of short motifs. Optimal alignment is sought in both forward and reverse motif directions.

*Multiple-motif alignment.* Two multiple-alignment strategies are tested. One is a progressive profile alignment strategy, which relies on the preconstruction of an approximate guide tree using UPGMA. The multiple alignment is built up by progressively aligning the nodes on the guide tree in order of decreasing similarity. In this way, each internal node contains a “familial” profile. The alignment at the root node will represent the final multiple alignment. The second implemented strategy is *iterative refinement alignment,* which aims to combat the problem of local minima, common in the progressive alignment methods due to “frozen” subalignments [43]. Iterative refinement builds a rough multiple alignment by progressively adding to the current alignment the most similar input PSSM (and taking the most similar pair as the start point of the multiple alignment). Once the initial alignment is built, each PSSM is removed from the alignment in turn and realigned to a profile of the other aligned sequences. Iteration of the realignment continues a fixed number of times. For gapped multiple alignments, gaps are encouraged to open in the same positions as previous gaps by negatively weighting the gap-open and gap-extend penalties in positions of the alignment that already contain gaps.

*Tree-building algorithms.* We implemented two similarity-based tree-building algorithms: an agglomerative method (UPGMA [44]) and a divisive method that is based on a self-organizing tree algorithm (SOTA [45]). UPGMA begins by assigning each input PSSM its own leaf node. At each timestep, the two nodes with the maximum average pairwise similarity are joined. The tree is built up through successive combinations of nodes until only one node (the root) remains. SOTA follows the opposite strategy. The tree is initialized with only one node (the root), which contains a rough alignment of all input PSSMs, and the node model is generated from this alignment. The root node then produces two identical offspring leaf nodes. During each timestep, the algorithm assigns the PSSMs to their most similar leaf nodes and then allows the node model to be updated in accordance with their current contents. As is characteristic of self-organizing neural algorithms, SOTA also allows for small contributions from neighboring nodes during the update step. These contributions are designed to keep neighboring nodes similar. After a number of timesteps, the node with the highest degree of dissimilarity amongst its members is allowed to produce two identical offspring nodes. This competitive learning scheme continues until each leaf node contains a single PSSM. While we denote this algorithm SOTA to reflect its similarity to the concepts presented by Dopazo and Carazo [45], the neural tree algorithm implemented here may also be thought of as a weighted hierarchical binary *k*-means algorithm.

*Estimating the number of data clusters in a PSSM tree.* In this study, we tested two known statistics: Hastie, Tibshirani, Walter (*HTW*) [22] and Calinski and Harabasz (*CH*) [19], and one derivative of the latter (Table 4). For the given dataset, we found that *CH*_log_ performed the best. The *CH*_log_ formula is given in Table 4, with *B* and *W* representing the between and within cluster sum of squared errors, *n* is the number of matrices (i.e., 71 for the JASPAR dataset), and *g* is the current number of clusters in the hierarchy. Error here refers to the negative logarithm of the motif comparison *p*-value (described above). In contrast to the original Calinski and Harabasz statistic, the *B* and *W* are log-transformed to avoid the tendency of the denominator towards zero, which results from the relatively small size of the datasets in which we operate. This logarithm transformation means that the estimate for the optimal number of clusters on the tree (*K*) is the value of *g* that minimizes *CH*_log_ (in the original Calinski and Harabasz formula, the maximum value of *CH*_log_ was sought). The suitability of the above metric to the current application domain was confirmed by evaluating its performance in a dataset with a small number of tightly clustered motif groups (see Results).

#### Motif datasets and structures.

For the purposes of testing the accuracy of various motif-alignment and tree-building strategies, we compiled two datasets that contain the motifs of the families with four or more profiles in JASPAR [18] and TRANSFAC (release 9.3 [17]) databases. JASPAR contained 96 such motifs in 13 such families (of which 25 motifs belong to three zinc finger families). TRANSFAC contained 586 motifs in 25 families (of which 160 motifs belong to five zinc-finger families). The edges of the motifs were “trimmed” down to the first position with information content 0.4 or more.

## Supporting Information

Table S1 Relative Performance of All Tested Motif Alignment Strategies Performance is measured as the percent of motifs whose structural class are correctly recovered via the best hit in database searches. The two datasets used in this comparison are taken from JASPAR and TRANSFAC. While accuracy was measured over the complete dataset, the results below report separately the performance for the nonzinc-finger families (non-ZNF; ten families in JASPAR, 20 in TRANSFAC) and the zinc-finger families (ZNF; three in JASPAR, five in TRANSFAC). *Average* is the weighted average. Overlapping (*overlap*) and ungapped (*ungapped*) alignments are specified. Gap extension is equal to half the gap opening.
(243 KB DOC)

### Accession Numbers

The structures we used in Figure 8 have the following Protein Data Bank (http://www.pdb.org) accession numbers: 9ANT, 2EZD, 1HRY, 1IF1, 1DH3, 1BC8, 2NLL, 1H88, 1PDN, 1SVC, 1SRS, 1MDY, 1AN2. The HNF3_Mod structure was provided by Kirk L. Clark and Stephen K. Burley (personal communication).

## Abbreviations

AKL: average Kullback–Leibler (metric)
ALLR: average log-likelihood ratio (metric)
C/EBP: CCAAT/enhancer binding protein
CREB: cAMP response element-binding
FBP: familial binding profile
HMG: high mobility group
LOOCV: leave-one-out cross-validation
PCC: Pearson's correlation coefficient (metric)
pCS: *p*-value of chi-square (metric)
PSSM: position-specific scoring matrix
SOTA: self-organizing tree algorithm
SSD: sum of squared distances (metric)
STAMP: similarity, tree-building, and alignment of DNA motifs and profiles
TF: transcription factor
UPGMA: unweighted pair group method with arithmetic mean
