# Supplementary material for: DNA Familial Binding Profiles Made Easy: Comparison of Various Motif Alignment and Clustering Strategies
Source: PLoS Comput Biol. 2007 Mar 30;3(3):e61. doi: 10.1371/journal.pcbi.0030061 (PMC1848003; doi:10.1371/journal.pcbi.0030061)
Supplement: Table S1 — Performance is measured as the percent of motifs whose structural class are correctly recovered via the best hit in database searches. The two datasets used in this comparison are taken from JASPAR and TRANSFAC. While accuracy was measured over the complete dataset, the results below report separately the performance for the nonzinc-finger families (non-ZNF; ten families in JASPAR, 20 in TRANSFAC) and the zinc-finger families (ZNF; three in JASPAR, five in TRANSFAC). Average is the weighted average. Overlapping (overlap) and ungapped (ungapped) alignments are specified. Gap extension is equal to half the gap opening. (243 KB DOC) [file pcbi.0030061.st001.doc]

**Supplementary Table 1:** Relative performance of all tested motif alignment strategies. Performance is measured as the percent of motifs whose structural class are correctly recovered *via* the best-hit in database searches. The two datasets used in this comparison are taken from JASPAR and TRANSFAC. While accuracy was measured over the complete dataset, the results below report separately the performance for the non zinc-finger families (non-ZNF; 10 families in JASPAR, 20 in TRANSFAC) and the zinc-finger families (ZNF; 3 in JASPAR, 5 in TRANSFAC). ***Avg*** is the weighted average. Overlapping (***ovrlp***) and ungapped (***ungap***) alignments are specified. Gap extension is equal half the gap opening.

| **Alignment algorithm** | **Similarity metric** | **Gap open** | **JASPAR non-ZNF** | **JASPAR ZNF** | ***TRANSFAC non-ZNF*** | ***TRANSFAC ZNF*** | ***Avg.*** |
| --- | --- | --- | --- | --- | --- | --- | --- |
| SW | SSD | 1.00 | 0.845 | 0.480 | 0.833 | 0.788 | **0.811** |
| SW (ovrlp) | SSD | 1.00 | 0.845 | 0.480 | 0.833 | 0.788 | **0.811** |
| SW | SSD | 0.75 | 0.845 | 0.440 | 0.831 | 0.794 | **0.809** |
| SW (ovrlp) | SSD | 0.75 | 0.845 | 0.440 | 0.831 | 0.794 | **0.809** |
| SW | SSD | 0.50 | 0.845 | 0.520 | 0.824 | 0.794 | **0.808** |
| SW (ovrlp) | SSD | 0.50 | 0.845 | 0.520 | 0.824 | 0.794 | **0.808** |
| SW | PCC | 1.50 | 0.845 | 0.520 | 0.817 | 0.800 | **0.805** |
| SW | SSD | 0.25 | 0.859 | 0.560 | 0.808 | 0.806 | **0.804** |
| SW (ovrlp) | SSD | *0.25* | 0.859 | 0.560 | 0.808 | 0.806 | **0.804** |
| SW | PCC | 1.00 | 0.831 | 0.600 | 0.815 | 0.788 | **0.802** |
| SW (ovrlp) | PCC | 1.00 | 0.831 | 0.600 | 0.812 | 0.788 | **0.801** |
| SW (ungap) | PCC | N/A | 0.887 | 0.600 | 0.812 | 0.763 | **0.801** |
| SW | PCC | 1.50 | 0.845 | 0.520 | 0.810 | 0.800 | **0.801** |
| NW | SSD | 1000 | 0.859 | 0.480 | 0.817 | 0.781 | **0.801** |
| SW | SSD | 1000 | 0.859 | 0.480 | 0.817 | 0.781 | **0.801** |
| SW (ovrlp) | SSD | 1000 | 0.859 | 0.480 | 0.817 | 0.781 | **0.784** |
| SW | SSD | 2.00 | 0.859 | 0.480 | 0.817 | 0.781 | **0.784** |
| SW (ovrlp) | SSD | 2.00 | 0.859 | 0.480 | 0.817 | 0.781 | **0.784** |
| SW | PCC | 2.00 | 0.845 | 0.520 | 0.812 | 0.788 | **0.783** |
| SW | PCC | 0.50 | 0.845 | 0.600 | 0.812 | 0.769 | **0.791** |
| SW (ovrlp) | PCC | 0.50 | 0.845 | 0.600 | 0.812 | 0.769 | **0.791** |
| SW (ovrlp) | PCC | 2.00 | 0.831 | 0.520 | 0.810 | 0.794 | **0.778** |
| SW | PCC | 1000 | 0.817 | 0.520 | 0.812 | 0.788 | **0.773** |
| SW | PCC | 4.00 | 0.817 | 0.520 | 0.812 | 0.788 | **0.773** |
| SW (ovrlp) | PCC | 4.00 | 0.817 | 0.520 | 0.805 | 0.800 | **0.772** |
| NW | PCC | 1000 | 0.817 | 0.520 | 0.805 | 0.794 | **0.771** |
| SW (ovrlp) | PCC | 1000 | 0.817 | 0.520 | 0.805 | 0.794 | **0.771** |
| SW (ovrlp) | KL | 4.00 | 0.845 | 0.480 | 0.789 | 0.800 | **0.771** |
| NW | KL | 1000 | 0.831 | 0.440 | 0.796 | 0.781 | **0.760** |
| SW (ovrlp) | KL | 2.00 | 0.831 | 0.480 | 0.791 | 0.788 | **0.765** |
| SW | KL | 1000 | 0.831 | 0.440 | 0.798 | 0.775 | **0.760** |
| SW (ovrlp) | KL | 1000 | 0.831 | 0.440 | 0.793 | 0.788 | **0.760** |
| SW (ovrlp) | KL | 1.50 | 0.831 | 0.480 | 0.786 | 0.788 | **0.763** |
| SW | KL | 1.50 | 0.845 | 0.400 | 0.779 | 0.781 | **0.755** |
| SW (ovrlp) | KL | 1.00 | 0.817 | 0.480 | 0.779 | 0.775 | **0.754** |
| SW | KL | 1.00 | 0.859 | 0.440 | 0.768 | 0.781 | **0.761** |
| NW | ALLR_LL | 2.00 | 0.859 | 0.400 | 0.791 | 0.719 | **0.755** |
| NW | SSD | 0.25 | 0.831 | 0.560 | 0.765 | 0.775 | **0.764** |
| SW | KL | 0.50 | 0.831 | 0.520 | 0.758 | 0.769 | **0.756** |
| SW (ovrlp) | KL | 0.50 | 0.817 | 0.400 | 0.756 | 0.775 | **0.735** |
| SW | KL | 4.00 | 0.831 | 0.400 | 0.772 | 0.719 | **0.738** |
| NW | ALLR_LL | 3.00 | 0.845 | 0.440 | 0.756 | 0.744 | **0.746** |
| NW | KL | 0.50 | 0.817 | 0.440 | 0.744 | 0.769 | **0.735** |
| NW | KL | 1.50 | 0.789 | 0.520 | 0.737 | 0.781 | **0.734** |
| NW | SSD | 0.50 | 0.746 | 0.640 | 0.754 | 0.731 | **0.733** |
| SW | KL | 2.00 | 0.831 | 0.520 | 0.742 | 0.738 | **0.745** |
| NW | KL | 1.00 | 0.803 | 0.400 | 0.739 | 0.769 | **0.723** |
| NW | ALLR | 2.00 | 0.775 | 0.400 | 0.754 | 0.713 | **0.710** |
| NW | ALLR_LL | 1000 | 0.746 | 0.280 | 0.756 | 0.719 | **0.685** |
| SW (ovrlp) | ALLR_LL | 1000 | 0.746 | 0.280 | 0.756 | 0.719 | **0.685** |
| SW (ovrlp) | ALLR_LL | 3.00 | 0.732 | 0.320 | 0.756 | 0.713 | **0.685** |
| SW (ovrlp) | ALLR_LL | 4.00 | 0.746 | 0.280 | 0.754 | 0.719 | **0.685** |
| NW | ALLR_LL | 4.00 | 0.817 | 0.480 | 0.723 | 0.725 | **0.726** |
| SW (ovrlp) | ALLR_LL | 2.00 | 0.761 | 0.360 | 0.749 | 0.700 | **0.696** |
| NW | KL | 2.00 | 0.746 | 0.400 | 0.725 | 0.756 | **0.695** |
| SW | ALLR_LL | 1000 | 0.775 | 0.320 | 0.739 | 0.713 | **0.694** |
| SW | ALLR_LL | 4.00 | 0.761 | 0.320 | 0.744 | 0.700 | **0.689** |
| SW (ovrlp) | pCS | 0.75 | 0.761 | 0.560 | 0.718 | 0.725 | **0.714** |
| NW | PCC | 0.50 | 0.761 | 0.640 | 0.718 | 0.713 | **0.723** |
| SW (ovrlp) | pCS | 2.00 | 0.732 | 0.600 | 0.723 | 0.713 | **0.709** |
| SW (ovrlp) | pCS | 1.00 | 0.761 | 0.560 | 0.718 | 0.719 | **0.713** |
| NW | ALLR_LL | 1.00 | 0.831 | 0.360 | 0.735 | 0.663 | **0.712** |
| SW (ovrlp) | ALLR | 5.00 | 0.662 | 0.320 | 0.746 | 0.713 | **0.655** |
| SW (ovrlp) | pCS | 1000 | 0.732 | 0.600 | 0.721 | 0.706 | **0.707** |
| SW (ovrlp) | pCS | 1000 | 0.732 | 0.600 | 0.721 | 0.706 | **0.707** |
| SW | ALLR_LL | 3.00 | 0.732 | 0.320 | 0.739 | 0.694 | **0.676** |
| SW (ungap) | SSD | N/A | 0.775 | 0.560 | 0.714 | 0.700 | **0.714** |
| SW (ovrlp) | ALLR | 2.00 | 0.718 | 0.320 | 0.735 | 0.694 | **0.669** |
| SW (ungap) | pCS | N/A | 0.690 | 0.400 | 0.742 | 0.675 | **0.669** |
| NW | ALLR | 1000 | 0.676 | 0.320 | 0.735 | 0.706 | **0.655** |
| SW (ovrlp) | ALLR | 10.00 | 0.662 | 0.320 | 0.737 | 0.706 | **0.651** |
| SW (ovrlp) | ALLR | 1000 | 0.662 | 0.320 | 0.737 | 0.706 | **0.651** |
| SW (ovrlp) | ALLR | 15.00 | 0.662 | 0.320 | 0.737 | 0.706 | **0.651** |
| SW (ovrlp) | ALLR | 20.00 | 0.662 | 0.320 | 0.737 | 0.706 | **0.651** |
| SW | ALLR | 5.00 | 0.704 | 0.320 | 0.732 | 0.694 | **0.663** |
| SW (ovrlp) | pCS | 0.50 | 0.718 | 0.600 | 0.711 | 0.694 | **0.697** |
| SW | ALLR_LL | 2.00 | 0.732 | 0.320 | 0.728 | 0.688 | **0.671** |
| SW | ALLR | 2.00 | 0.704 | 0.320 | 0.732 | 0.688 | **0.662** |
| SW | ALLR | 10.00 | 0.718 | 0.320 | 0.721 | 0.694 | **0.664** |
| SW | ALLR | 1000 | 0.718 | 0.320 | 0.721 | 0.688 | **0.663** |
| SW | ALLR | 15.00 | 0.718 | 0.320 | 0.721 | 0.688 | **0.663** |
| SW | ALLR | 20.00 | 0.718 | 0.320 | 0.721 | 0.688 | **0.663** |
| SW (ovrlp) | ALLR_LL | 1.00 | 0.789 | 0.360 | 0.707 | 0.663 | **0.686** |
| SW | ALLR_LL | 1.00 | 0.704 | 0.360 | 0.714 | 0.656 | **0.656** |
| NW | SSD | 0.75 | 0.718 | 0.560 | 0.700 | 0.644 | **0.681** |
| SW (ungap) | KL | N/A | 0.746 | 0.480 | 0.676 | 0.688 | **0.678** |
| SW (ovrlp) | pCS | 0.25 | 0.662 | 0.600 | 0.685 | 0.669 | **0.663** |
| SW (ungap) | ALLR_LL | N/A | 0.775 | 0.280 | 0.683 | 0.644 | **0.659** |
| SW (ungap) | ALLR | N/A | 0.775 | 0.320 | 0.676 | 0.656 | **0.663** |
| NW | PCC | 1.00 | 0.690 | 0.680 | 0.664 | 0.663 | **0.676** |
| NW | pCS | 0.75 | 0.662 | 0.600 | 0.657 | 0.675 | **0.660** |
| NW | pCS | 1.00 | 0.662 | 0.440 | 0.653 | 0.688 | **0.654** |
| NW | pCS | 0.50 | 0.690 | 0.560 | 0.653 | 0.650 | **0.652** |
| NW | pCS | 0.25 | 0.634 | 0.640 | 0.650 | 0.656 | **0.650** |
| NW | ALLR | 5.00 | 0.634 | 0.480 | 0.648 | 0.619 | **0.633** |
| NW | AKL | 4.00 | 0.704 | 0.400 | 0.594 | 0.600 | **0.600** |
| NW | pCS | 2.00 | 0.563 | 0.400 | 0.596 | 0.656 | **0.600** |
| NW | SSD | 1.00 | 0.634 | 0.480 | 0.585 | 0.581 | **0.585** |
| NW | PCC | 1.50 | 0.606 | 0.520 | 0.580 | 0.581 | **0.581** |
| NW | PCC | 2.00 | 0.521 | 0.480 | 0.423 | 0.500 | **0.453** |
| NW | ALLR | 10.00 | 0.394 | 0.440 | 0.343 | 0.400 | **0.365** |
| NW | SSD | 2.00 | 0.437 | 0.400 | 0.324 | 0.375 | **0.350** |
| NW | ALLR | 15.00 | 0.268 | 0.400 | 0.261 | 0.381 | **0.295** |
| NW | PCC | 4.00 | 0.282 | 0.480 | 0.282 | 0.269 | **0.286** |
| NW | ALLR | 20.00 | 0.268 | 0.440 | 0.232 | 0.306 | **0.261** |
